# Cardiovascular Clinical Assessment in Greyster Dogs in Bikejöring Training

**DOI:** 10.3390/ani10091635

**Published:** 2020-09-11

**Authors:** Mila Benito, Laure Boutigny

**Affiliations:** 1 Department of Animal Medicine and Surgery, Canine Sports Medicine Service, Veterinary School, Universidad Cardenal Herrera-CEU, 46115 Valencia, Spain; 2 DVM, 88220 Hadol, France; lboutigny@gmail.com

**Keywords:** canine athlete adaptation, bikejöring, echocardiography, Greyster, doppler, dryland mushing, cardiovascular response

## Abstract

**Simple Summary:**

Regular intense exercise is known to induce cardiac hypertrophy in some dogs engaged in different physical activities, but the cardiovascular response in dogs that routinely carry out aerobic exercise in the form of bikejöring (a form of dryland mushing) is unknown. The pre- and post-competition clinical examinations usually carried out in canine athletes include an electrocardiogram due to its diagnostic value, but some cardiac structural disturbances may go unnoticed at rest, especially in the early stages of disease. In our study, changes in systolic, mean and pulse pressure were detected after exercise, while diastolic blood pressure remained stable. Numerous changes in echocardiographic variables (LVFS-left ventricle fractional shortening, LVEF-left ventricle ejection fraction, EPSS-E-point to septal separation, CO-cardiac output, CI-cardiac index, PWd-posterior wall thickness at end-diastole and major/minor axis ratio) were also found. No association was found between the sex of the animals and the differences in the data. Our findings lead us to recommend theLVF introduction of echocardiograms for the clinical evaluation of canine athletes competing in this form of mushing. Furthermore, by including echocardiograms in clinical examinations during physical training, knowledge of the individual cardiovascular response after exercise can be improved.

**Abstract:**

Bikejöring is a type of dryland mushing requiring high-intensity aerobic effort, with speed peaks close to 42 km/h. Greysters (crosses between the German Shorthaired Pointer and the Greyhound) often participate in such events and perform well. The objective of this comparative study was to evaluate the clinical use of non-invasive methods in assessing the cardiovascular health of 22 Greyster dogs in physical training, by determining the differences between different cardiovascular parameters before and after physical training. Blood pressure, heart rate and echocardiographic results were compared. The mean age of the dogs was 4.4 years ± 1.8% and 54.5% were female. All participating dogs regularly participated in bikejöring. Post-exercise increases were observed in systolic blood pressure (SBP), mean arterial pressure (MBP) and pulse pressure (SBPD), with diastolic blood pressure (DBP) remaining stable. Changes of clinical interest were observed in numerous echocardiographic variables such as left ventricle fractional shortening (LVFS), left ventricule ejection fraction (LVEF), E-point to septal separation (EPSS), cardiac output (CO), cardiac index (CI), posterior wall thickness at end-diastole (PWd) and major/minor axis ratio (MA/ma), including a decrease in the shortening fraction and an increase in EPSS after exercise. These clinical findings were observed in both males and females; they do not appear to be associated with dilated cardiomyopathy, but rather with a cardiovascular response to physical training. This study derives from the real interest of clinical veterinarians who care for highly trained canine athletes. It contributes to an increase in knowledge of the different cardiac adaptations of such dogs after intense exercise and serves to differentiate these from pathologic conditions.

## 1. Introduction

Regular intense exercise is known to be able to induce cardiac hypertrophy in dogs engaged in different physical activities, such as that of search and rescue [1,2] or sporting activities such as agility or mushing. Numerous studies have been carried out on Alaskan sled dogs, demonstrating the cardiac remodeling that these dogs present over time due to such activity [3,4].

In recent years, there has been an increase in the participation of canine athletes in other types of mushing, taking place on dry land rather than snow. One of these, bikejöring, has significantly increased in popularity in Central European countries, with optimal weather conditions being present there for this sport. In this discipline, cyclists are assisted by one or two dogs which pull the bicycle along and there are organized races which may be sprints (up to eight kilometers) or ones of medium distance (approximately 30 km).

In these competitions, the Greyster is a popular breed and yet this is not one of those typically studied in the literature, which has primarily focused on sled dogs running on snow. These dogs were originally bred in Norway in the 1980s, from a series of crossings between the German Shorthaired Pointer and the Greyhound, for the original purpose of participation in sledging races. As with other such breeds, they have been adapted and selected for their occupation through improved endurance, cardiac function, blood flow and cognitive performance [5].

Greysters have been shown to be highly competitive mushing dogs, demonstrating great athletic performance. There are no data in the scientific literature regarding exercise-induced hemodynamic changes in Greyster dogs, but a high degree of training would be expected to lead to altered echocardiographic values in these dogs in comparison with the usual values for sedentary dogs. These echocardiographic changes could make these dogs appear to have cardiomyopathies, which could carry a risk in competition. Given that, in the pre-competition examination, the veterinarians attending the races do not have enough time to individually evaluate each of the participating dogs, we propose the performance of a cardiovascular clinical study in the field, imitating the real conditions in order to study cardiovascular changes in physical training.

The objective of this comparative study was therefore to perform a clinical evaluation of different cardiovascular parameters before and after physical training of 22 Greyster dogs, using non-invasive methods.

## 2. Materials and Methods

### 2.1. Animals

Healthy adult Greyster dogs (>18 months of age) who regularly take part in bikejöring (minimum 3 h/week) were included in this study. One dog initially included had to be excluded due to a recent injury, so 22 dogs were ultimately enrolled on the full study. All of them were of the same breed and engaged in the same sporting activity. The study was conducted on the same day for all dogs, during the final phase of the sports season. The dogs belonged to a total of twelve owners, with two of them owning 13 of the dogs, more than half of the sample. All the dogs were from the same geographical area, with the same orography and similar training systems.

The physical training consisted of completing an identical route of 4.37 km. Each dog wore a sports harness and was attached to the owner’s bicycle (or “musher”) by a shooting line. The equipment is approved by the International Federation of Sleddog Sports for this sport.

All records were taken without sedation or restrictions. The owner was always with the dog and the animals were allowed a period of time (less than 1 min) to adapt to the procedures for taking blood pressure, heart rate and echocardiography to minimize the effect of excitation.

Post-exercise measurements were made individually, over three consecutive days with temperatures ranging from 0 °C to 5 °C. Average speed and maximum speed during exercise session were recorded for each pair (dog-musher). A maximum speed of 42.6 km/h was reached in the 4.37 km race.

### 2.2. Blood Pressure Measurement

For the measurement of blood pressure, an Omron^®^ RS6 automatic blood pressure monitor was used, whose length covers 80% of the perimeter of the thoracic limb of these dogs.

For the pre-exercise measurement (T0), we waited three minutes after fitting the monitor before taking the measurement. The sphygmomanometer was placed on the left forelimb, near the elbow joint, on the superficial brachial artery. The post-exercise blood pressure measurement (T1) was performed immediately after the exercise was completed.

### 2.3. Electrocardiographic Recordings

One of the veterinarians devoted herself exclusively to performing the electrocardiographic recordings, before (T0) and immediately after exercise (T1). ECGs were recorded and analyzed with an SRM^®^ CardioScout three-channel electrocardiograph connected via Bluetooth with the associated SRM^®^ software. The electrocardiogram was performed on each dog on the right lateral decubitus and the electrodes were attached to the skin folds using suitable forceps, and the areas of the skin in contact with the electrodes were impregnated with an alcohol solution. The electrodes were fixed on the palmar surface of the right and left thoracic limbs at the olecranon and on the internal surface of the groin. A six-lead baseline ECG recording at 25 mm·sg^−1^ and with a calibration of 0.1 mV·mm^−1^ was obtained. The rhythm and heart rate at rest, the electrical axis, the amplitude and duration of the P, Q and R waves and the QRS complex were analyzed, as well as the duration of the PR and QT intervals. Checks for possible conduction disturbances were made.

### 2.4. Echocardiogram

A transthoracic echocardiographic study was also performed before (T0) and after (T1) exercise, using a Sonosite^®^ EDGE II portable device. The following parameters were evaluated in TM-mode in the right parasternal window on the transverse axis: the interventricular septum thickness at end-diastole (IVSd) and end-systole (IVSs), the diameter of the left internal ventricle in diastole (LVIDd) and systole (LVIDs), the left ventricle free wall in diastole (PWd-posterior wall thickness at end-diastole) and systole (PWs-posterior wall thickness at end-systole) and the separation of the septum from point E (EPSS).

The device automatically calculated the shortening fraction (LVFS) and the ejection fraction (LVFE-left ventricle ejection fraction). The diameter of the left atrium (LA) and the aorta (Ao) was calculated in mode B. The continuous doppler mode was selected for the assessment of the left ventricular outflow (LVOT). Finally, color Doppler enabled a visual assessment to be performed of the presence of abnormal flows in systoles in the mitral and aortic valve area. All echocardiographic studies were performed with the dogs in the right lateral decubitus position.

### 2.5. Data Analysis

A descriptive statistical analysis was carried out, with means and standard deviations being recorded and inferential statistical tests, such as independent *t*-tests, paired *t*-tests and Pearson’s correlation coefficient, were performed, using the IBM SPSS Statistics 24 software. First, the relationship between sex and all the blood pressure, heart rate and echocardiographic variables was tested, before and after exercise, in order to determine if the sample could be studied as a single group. Paired *t*-tests were carried out to determine if there were statistical differences between the pre- and post-exercise blood pressure, heart rate and echocardiographic variables. Finally, Pearson’s correlation coefficient was calculated for specific variables.

## 3. Results

Before starting the exercise training, body data and measurements were obtained from all 22 dogs, 12 females (54.5%) and 10 males (45.5%). The mean age was 4.4 years ± 1.8. Zoometric measurements such as height at the withers (70.6 cm ± 5.4) and thoracic perimeter (73.5 cm ± 4.7) were recorded. All the animals were weighed, with the mean weight being 29.9 kg ± 5.5.

All teams (musher and dog) completed the exercise challenges without difficulties or abnormal symptoms.

The results of the complementary tests are shown below in the order in which they were performed On rare occasions, some specific measurements do not appear in tables, either because the parameter was not properly recorded or due to technical failure. In such cases, the data are counted as missing, invalidating that animal for that specific measurement. In this respect, it is important to note that it such measurement errors did not always occur with the same dog (which would have enabled us to exclude this dog from the study).

### 3.1. Blood Pressure

#### 3.1.1. Descriptive Statistical Analysis

A descriptive analysis of the variables related to blood pressure was carried out, with their mean values, standard deviations and the minimum and maximum values being recorded.

This analysis was undertaken for sample as a whole without taking sex into account, since, as is discussed below, no significant differences were found between any of the variables in the three sections (blood pressure, heart rate and echocardiography) with regard to the sex of the animals.

The values obtained were, before and after exercise, respectively: for heart rate, measured in beats per minute, 104.1 ± 22 and 153.1 ± 27.5; for systolic blood pressure, 115.7 mm Hg ± 20.6 and 129.5 mm Hg ± 17.8; for diastolic blood pressure, 76 mm Hg ± 14.9 and 76.9 mm Hg ± 14.3; and for mean blood pressure, 64.5 mm Hg ± 11 and 94.4 mm Hg ± 14.3.

#### 3.1.2. Independent *t*-Test

An independent *t*-test was performed to determine the existence of sex differences for the variables of heart rate (HR), systolic blood pressure (SBP), diastolic blood pressure (DBP), mean arterial pressure (MBP) and pulse pressure (SBPD), before and after exercise. As can be seen Table 1, no significant differences between the sexes were found and, therefore, in the subsequent tests, the 22 dogs were considered as a single group.

#### 3.1.3. Pearson’s Correlation Coefficient

The Pearson’s correlation coefficient between the heart rate and systolic blood pressure was calculated before and after exercise (see Table 2), obtaining significant results only in the case of the pre-exercise values, where it was found that the higher the heart rate, the greater the systolic pressure.

#### 3.1.4. Paired *t*-Test

Four mean difference tests were performed for related samples of the following variables: HR, SBP, DBP, MBP and SBPD, before and after exercise.

Table 3 show that significant differences were found between the before and after exercise variables, except in the case of diastolic pressure. All the HR, SBP, MBP and SBPD values were significantly higher post-exercise.

### 3.2. Electrocardiographic Variables

#### 3.2.1. Descriptive Statistical Analysis

A descriptive analysis of the electrocardiographic variables was performed, and their mean values and standard deviations recorded. The mean pre- and post-exercise values were, respectively: for the amplitude (mV) of the R wave, 2.5 ± 0.79 and 2.67 ± 0.80; for the Q wave, 0.83 ± 0.40 and 0.74 ± 0.36; and for the QT interval duration (ms), 0.21 ± 0.01 and of 0.18 ± 0.02.

#### 3.2.2. Independent *t*-Test

An independent *t*-test was performed to determine the existence of sex differences for the HR, R Wave, Q Wave and QT Interval variables, before and after exercise. As can be seen in Table 4, no significant differences were found between the sexes and, therefore, in the subsequent tests, the 22 dogs were considered as a single group.

#### 3.2.3. Pearson’s Correlation Coefficient

Four Pearson’s correlation coefficients were calculated: for the QT interval and the VS, both pre- and post-exercise and for the QT interval and heart rate, also both pre- and post exercise. A significant correlation was only found between the post-exercise QT interval and heart rate, as can be seen in Table 5, demonstrating that the lower the heart rate, the longer the QT interval.

#### 3.2.4. Paired *t*-Test

For the pre- and post-exercise values for the R waves, Q waves and the QT intervals, a significant difference was not found in the case of the Q wave but was present in the other two cases (see Table 6). For the R wave, its value was significantly higher after exercise, whereas for the QT interval, its pre-exercise value was significantly higher.

### 3.3. Echocardiography

#### 3.3.1. Descriptive Statistical Analysis

A descriptive analysis of the echocardiographic variables was performed and their mean values, standard deviations and the minimum and maximum values of each of them are in Table 7 below.

#### 3.3.2. Independent *t*-Test

As in the previous sections, an independent *t*-test was performed to determine the existence of sex differences for the following echocardiographic variables: LVFS, LVFE, EPSS, LA/AO, MA/ma, SV, CO, CI, LVIDd, LVIDs, PWd, PWs, IVSd, IVSs, LVOT. As shown in Table 8, no significant differences were found, as with the other cases.

#### 3.3.3. Paired *t*-Test

The pre- and post-exercise ultrasound variables were compared with paired *t*-tests and significant differences were found in the cases of LVFS, LVFE, EPSS, MA/ma, CO, CI and PWd. Thus, the left ventricular shortening fraction (LVFS) and the left ventricular ejection fraction (LVFE) present lower post-exercise values (see Table 9), whereas these are higher for EPSS, as is the ratio between the major axis and the minor axis (MA/ma). As expected, cardiac output and the cardiac index are higher post-exercise and PWd also presents statistically significant higher values after exercise.

## 4. Discussion

The objective of this study was to investigate variations in blood pressure, electrocardiographic and echocardiographic variables before and after exercise in 22 Greyster dogs under real conditions.

Before the full battery of pre and post-exercise analyses were performed, independent *t*-tests were carried out to determine if sex differences existed in the sample, but none were found and so the entire sample was analyzed as a single group.

Regarding the blood pressure variables, a significant correlation between heart rate and systolic pressure was found. Systolic blood pressure increases during progressive exercise as part of the necessary increase in cardiac output and oxygen consumption, while diastolic blood pressure remains relatively stable. Aerobic exercise in these trained and healthy dogs therefore produces a considerable increase in pulse pressure [6]. This results in a decrease in general peripheral resistance, which guarantees greater blood supply and better oxygen supply for the tissues doing the work, mainly the muscles, enabling them to function more economically and effectively [7].

Regarding the pre- and post-exercise differences, as expected, higher post-exercise values were found in SBP, MBP and SBPD. This is due to hemodynamic adaptation, affecting blood pressure and peripheral resistance. With exercise, systolic pressure increases with cardiac output, without diastolic changes, probably due to the drop in peripheral vascular resistance due to the activation of cellular chemoreceptors. Heart rate also increases due to reduced vagal tone and increased sympathetic tone, resulting in elevated systolic blood pressure [7].

Second, regarding the electrocardiographic variables, we found a single significant correlation between the QT interval and the heart rate after exercise. The changes in QT interval could be due to many factors: HR, heart size, autonomic tone, serum calcium and potassium concentrations, and the administration of drugs could influence the duration of cardiac action potentials. HR was a negative determinant of QT-interval duration [8]. The presence, therefore, of wider QT intervals before exertion is consistent with this correlation and can be explained by the effect of sympathetic hyperactivity.

In the case of the pre- and post-exercise amplitude values of the electrocardiographic waves, the R wave was found to be higher after exercise. The R wave represents the depolarization of both ventricular free walls from the subendocardial endings of the Purkinje fibers to the epicardium and the terminal depolarization of the bases of both ventricles and of the ventricular septa [9] whereas, in other canine sports activities, such as agility, the R wave voltage is lower [8] in sled dogs [10], but in Greyhounds [11] it is higher, suggesting larger external ventricular dimensions for sled dogs.

Regarding the Q wave, the Greyster dogs of this study did not present significant differences between males and females, although a somewhat higher pre-exercise voltage was observed in females. After exertion, however, a somewhat higher Q wave was observed in the males, although without significant differences. These appreciable amplitude increases in electrocardiography in Greyster dogs point to the need for echocardiographic studies to determine whether ventricular hypertrophy exists in these dogs taking part in bikejöring.

With respect to the echocardiographic values obtained, no significant differences were detected between males and females, and so all the dogs were studied as a single group, as with the blood pressure and electrocardiographic variables. Echocardiography has traditionally been used in veterinary clinics to measure the size of the atrial and ventricular chambers and so to predict the risk of congestive heart failure [12].There are thus numerous published studies that make reference to echocardiographic values and functional indices in canines [12,13,14,15,16,17,18,19,20,21,22,23,24], although to date there are no representative reference values for the entire canine species. Therefore, we do not have a reference bibliography for cardiovascular response to exercise in Greyster dogs.

The pre- and post-exercise echocardiographic values presented significant differences in several cases. The dogs in our study presented lower post-exercise shortening fraction (LVFS) values. In horses, a lower shortening fraction immediately after exercise has been associated with a decrease in physical performance [25]. This has not yet been described in canine athletes, and, therefore, there is a need to perform echocardiographic studies of these same dogs at rest and 30 min after exercise.

E-point-to-septal-separation (EPSS) is a valuable additional parameter for the diagnosis of dilated cardiomyopathy (CMD), enhancing the diagnostic capabilities of TM-mode and performing as well as the Sphericity Index [26]. EPSS has been suggested, in a study on Doberman Pinschers, as the most sensitive and specific criterion for early CMD, without showing overlap between healthy dogs and those with advanced CMD, thus demonstrating good sensitivity and specificity [26]. Calvert and Meurs proposed a cutoff value of > nine millimeters to diagnose CMD in this breed [27].

In our study, EPSS is significantly higher after exertion (10.13 mm; SD 0.25) and is also accompanied by an increase in the MA/ma ratio after exercise. Those results are consistent with the previous study [27] in that EPSS seems to be more sensitive than TM-mode (at least when using the published references for TM-mode). However, there is a discrepancy in the cutoff values for EPSS suggested above and this study, so studies with a larger sample need to be carried out in Greysters in order to demonstrate definitively that EPSS can be used objectively as a parameter of systolic function. However, it is reasonable to expect that—as in the human athlete [28]—the heart of the Greyster is also characterized by a global remodeling of the cavities, with a conserved biventricular systolic function or in the lower limit than normal. In any case, the higher EPSS of the dogs in our study does not decisively point to dilated cardiomyopathy, as one would expect this to be accompanied by decreased aortic blood flow, hypokinesis with fractional shortening (FS) < 25% and possible presentation of mitral/tricuspid regurgitation, aortic thrombosis, LVIDd > 46 mm, LVIDs > 38 mm and axis major-axis minor ratio < 1.5. In the resting state, therefore, the dogs under study did not present other indicators compatible with congestive heart failure due to dilated cardiomyopathy.

In addition, unsurprisingly, cardiac output was significantly higher post-exercise. This is due to cardiovascular changes, which occur at both the cardiac and peripheral levels. The first modification that dynamic exercise training such as bikejöring produces is an increase in the beating volume and, therefore, also in the cardiac output at a given heart rate. This is accompanied by an increase in end-diastolic volume as measured by echocardiography, which may be associated with a decrease in end-systolic volume, accompanied by an increase in the thickness of the ventricular wall [29].

The behavior of the aorta in sports dogs is currently unknown, but we found no significant differences for LVOT between males and females in 2D-mode, unlike those described in humans [29]. In human medicine, the aorta of athletes has not been found to follow the same adaptation pattern as other cardiac structures [30] and, therefore, if dilation is detected in a canine athlete, it could provide the clinical veterinarian with evidence of possible aortic or aortic valve disease. In the Greyster dogs in our study, no differences were found between males and females, and therefore, further studies with larger samples will be required to determine whether the aortic values in Greyster dogs change according to sex, sports activity and training.

Likewise, no significant differences were found in the LA/AO ratio before and after exercise. This ratio is useful to detect possible left atrial enlargement and predicts the risk of congestive heart failure [8]. In our study, we did not find any indicators of an enlarged LA, which can be suggestive of myxomatous mitral valve degeneration. Although it is thought that 10% of healthy dogs may reach a LA/AO value of 1.6 [31], none of the Greyster dogs reached this value, with mean pre- and post-exercise values being 1.10 and 1.11, respectively.

## 5. Conclusions

Intense physical exercise produces acute cardiovascular changes that can have an impact on the health and performance of canine athletes. In Greysters, this proposed exercise test provides useful information that enables their cardiovascular responses to be determined in real situations. No sex-based differences were observed in the pre- and post-exercise data, enabling us to consider the group as a whole. No changes in blood pressure were found which would indicate cases of hypertension. Immediately after exercise (bikejöring), the Greyster dogs presented changes in systolic pressure, while diastolic pressure remained stable. The electrocardiographic data indicated no pathologic changes. Finally, the echocardiographic data allowed us to improve our understanding of some of the morphologic and functional responses that Greyster dogs experiences by participating in the sport of bikejöring, adding to our knowledge of their normal values and enabling the assessment of possible cardiac damage after strenuous exercise.

Echocardiography, therefore, has shown itself to be an indispensable complementary test for the evaluation of structural or functional changes in the dogs studied, although reference intervals for transthoracic echocardiography in the Greyster are required.

## 6. Limitations of the Study

Given the difficulties in carrying out a stress test in dogs, the field test carried out in this study reveals some hemodynamic changes related to physical exercise. We have thus been able to record the echocardiographic, electrocardiographic and blood pressure changes produced after physical training in 22 Greyster dogs. However, more research is required to understand the influence of diet, training, and genetics in relation to hemodynamic changes, and so to fully understand the impact of exercise on the canine heart structure.

## Figures and Tables

**Table 1:** Independent *t*-test for sex differences in the blood pressure variables.

Variable	*t*	Df	Sig. (2-Tailed)
HR Pre	0.819	19	0.423
HR Post	0.744	20	0.466
SBP Pre	−0.495	19	0.626
SBP Post	−1.138	19	0.269
DBP Pre	−1.871	19	0.077
DBP Post	−0.883	19	0.388
MBP Pre	−1.100	19	0.285
MBP Post	−1.034	19	0.314
SBPD Pre	1.063	19	0.301
SBPD Post ^1^	−0.605	18.396	0.552

^1^ Equal variances not assumed for SBPD Post.

**Table 2:** Pearson’s correlation coefficient for HR (heart rate) and SBP (systolic blood pressure) before exercise.

		HR Pre	SBP Pre
HR Pre (bpm)	Pearson Correlation	1	0.509 ^1^
	Sig. (2-tailed)		0.018
	N	21	21
SBP Pre (mm Hg)	Pearson Correlation	0.509	1
	Sig. (2-tailed)	0.018	
	N	21	21
		**HR Post**	**SBP Post**
HR Post (bpm)	Pearson Correlation	1	0.275
	Sig. (2-tailed)		0.227
	N	22	21
SBP Post (mm Hg)	Pearson Correlation	0.275	1
	Sig. (2-tailed)	0.227	
	N	21	21

^1^ Significance taken to be 0.05 or lower (2-tailed).

**Table 3:** Paired *t*-test comparison of pre- and post-exercise blood pressure variables.

	Mean	SD	*t*	Df	Sig. (2-Tailed)
HR Pre–HR Post	−49.00000	31.65280	−7.094	20	0.000
SBP Pre–SBP Post	−13.20000	23.33193	−2.530	19	0.020
DBP Pre–DBP Post	−1.65000	21.24364	−0.347	19	0.732
MBP Pre–MBP Post	−30.20000	17.69805	−7.631	19	0.000
SBPD Pre–SBPD Post	−11.55000	12.94716	−3.990	19	0.001

**Table 4:** Independent *t*-test for sex differences in the electrocardiographic variables.

Variable	*t*	Df	Sig. (2-Tailed)
HR Pre	0.819	19	0.423
HR Post	0.744	20	0.466
R Wave Pre	−1.213	20	0.239
R Wave Post	−1.610	20	0.123
Q Wave Pre	−0.192	20	0.850
Q Wave Post	−0.759	20	0.457
QT Pre	−1.402	20	0.176
QT Post	−1.206	20	0.242

**Table 5:** Pearson’s correlation coefficient for the post-exercise QT Interval and HR.

		QT Post	HR Post
QT Post	Pearson’s R	1	−0.630 ^1^
	Sig. (bilateral)		0.002
	N	22	22
HR Post	Pearson’s R	−0.630	1
	Sig. (bilateral)	0.002	
	N	22	22

^1^ Correlation is significant at the 0.01 level (2-tailed).

**Table 6:** Paired *t*-test comparison of pre-and post-exercise electrocardiographic variables.

Variables	Mean	SD	*t*	Df	Sig. (2-Tailed)
R Wave Pre–R Wave Post	−0.20909	0.38161	−2.570	21	0.018
Q Wave Pre–Q Wave Post	0.09545	0.37857	1.183	21	0.250
QT Pre–QT Post	0.02455	0.01503	7.659	21	0.000

**Table 7:** Descriptive statistics for the pre- and post-exercise echocardiographic variables.

	N	Minimum	Maximum	Mean	SD
LVFS Pre	22	20.00	40.00	30.5150	5.93318
LVFS Post	21	17.00	39.00	26.2381	5.60272
LVFE Pre	22	33.00	71.00	55.8636	10.2274
LVFE Post	21	36.00	69.00	50.238	8.64071
EPSS Pre	22	0.51	1.19	0.8605	0.21729
EPSS Post	22	0.62	1.59	1.0132	0.25367
LA/Ao Pre ^1^	22	0.83	1.51	1.1027	0.17526
LA/Ao Post	22	0.76	1.37	1.1105	0.15579
MA/ma Pre	22	1.38	2.51	1.8155	0.29868
MA/ma Post	19	1.30	3.31	2.0116	0.40832
HR Pre	21	63.00	152.00	104.1429	22.04379
HR Post	22	88.00	198.00	153.1364	27.54787
CO Pre	21	0.60	4.50	2.2933	1.15288
CO Post	22	0.80	10.70	3.1818	2.57749
CI Pre	21	0.82	4.25	2.4010	1.07136
CI Post	22	0.84	11.50	3.7532	3.07920
SV Pre ^2^	22	40.70	110.30	78.5364	18.49024
SV Post	19	30.40	116.40	75.1368	23.65700
LVIDd Pre	22	4.54	7.08	5.3855	0.52650
LVIDd Post	21	3.63	6.98	5.3419	0.83366
LVIDs Pre	22	2.72	5.57	3.7891	0.58135
LVIDs Post	21	2.67	5.03	3.9576	0.69725
PWd Pre	22	0.76	1.70	1.1009	0.19275
PWd Post	21	0.79	1.79	1.1871	0.26371
PWs Pre	21	1.32	2.29	1.6210	0.26188
PWs Post	21	1.00	2.32	1.5871	0.30858
IVSd Pre	22	0.62	1.65	1.0618	0.24657
IVSd Post	22	0.67	1.53	1.0586	0.23443
IVSs Pre	21	1.19	2.71	1.6929	0.32728
IVSs Post	21	0.68	2.32	1.6462	0.39751
LVOT Pre	22	1.10	3.13	1.8723	0.41587
LVOT Post	22	1.13	2.84	1.9027	0.42996

^1^ LA/AO—left atrium to aorta ratio; ^2^ SV—stroke volume.

**Table 8:** Independent *t*-test for sex differences in the echocardiographic variables.

Variable	*t*	Df	Sig. (2-Tailed)
LVFS Pre	0.651	20	0.522
LVFS Post	0.396	19	0.696
LVEF Pre	0.149	20	0.883
LVEF Post	0.537	19	0.598
EPSS Pre	1.098	20	0.285
EPSS Post	0.184	20	0.856
LA/AO Pre	0.693	20	0.496
LA/AO Post	0.039	20	0.969
MA/ma Pre	−1.353	20	0.191
MA/ma Post	−1.684	17	0.110
HR Pre	0.819	19	0.423
HR Post	0.744	20	0.466
CO Pre	−0.121	19	0.905
CO Post	0.913	20	0.372
CI Pre	0.107	19	0.916
CI Post	0.394	20	0.698
SV Pre	−0.795	20	0.436
SV Post	−0.463	17	0.649
DVGd Pre	−0.903	20	0.377
DVGd Post	−0.348	19	0.732
DVGs Pre	−0.646	20	0.526
DVGs Post	−0.093	19	0.927
PPVId Pre	−1.856	20	0.078
PPVId Post ^1^	−1.533	10.690	0.154
PPVIs Pre	0.146	19	0.886
PPVIs Post ^1^	−1.303	10.884	0.219
SIVd Pre	−0.759	20	0.456
SIVd Post	−1.408	20	0.174
SIVs Pre	0.076	19	0.940
SIVs Post	−0.706	19	0.489
DTSIV Pre ^1^	−0.801	13.517	0.437
DTSIV Post	−1.534	20	0.141

^1^ Equal variances not assumed for PPVId Post, PPVIs Post, DTSIVpre.

**Table 9:** Paired *t*-test comparison between pre- and post-exercise echocardiographic variables.

	Mean	SD	*t*	Df	Sig. (2-Tailed)
LVFS Pre–LVFS Post	4.58714	8.11840	2.589	20	0.018
LVFE Pre–LVFE Post	5.76190	12.74325	2.072	20	0.050
EPSS Pre–EPSS Post	−0.15273	0.24312	−2.947	21	0.008
LA/AO Pre–LA/AO Post	−0.00773	0.27013	−0.134	21	0.895
MA/ma Pre–MA/ma Post	−0.21474	0.42962	−2.179	18	0.043
SV Pre–SV Post	2.24737	34.02598	0.288	18	0.777
CO Pre–CO Post	−0.91143	1.99808	−2.090	20	0.050
CI Pre–CI Post	−1.38476	2.82453	−2.247	20	0.036
LVIDd Pre–LVIDd Post	0.03857	0.94712	0.187	20	0.854
LVIDs Pre–LVIDs Post	−0.16143	0.62953	−1.175	20	0.254
PWd Pre–PWd Post	−0.11476	0.24867	−2.115	20	0.047
PWs Pre–PWs Post	0.02700	0.36339	0.332	19	0.743
IVSd Pre–IVSd Post	0.00318	0.23473	0.064	21	0.950
IVSs Pre–IVSs Post	0.05100	0.45321	0.503	19	0.621
LVOT Pre–LVOT Post	−0.03045	0.50676	−0.282	21	0.781

## Abbreviations

SBP—systolic blood pressure; DBP—diastolic blood pressure; MBP—mean blood pressure; SBPD—pulse pressure; LVFS—left ventricle fractional shortening; LVFE—left ventricle ejection fraction; EPSS—E-point to septal separation; LA—left atrium; LA/AO—left atrium to aorta ratio; MA/ma—major axis/minor axis ratio; SV—stroke volume; CO—cardiac output; CI—cardiac index; LVIDd—left ventricular internal dimension at end-diastole; LVIDs—left ventricular internal dimension at end-systole; PWd—posterior wall thickness at end-diastole; PWs—posterior wall thickness at end-systole; IVSd—interventricular septum thickness at end-diastole; IVSs—interventricular septum thickness at end-systole; LVOT—diameter of the left ventricular outflow tract; FA—fractional shortening; TM-mode—motion mode; HR—heart rate; SV—stroke volume; CMD—dilated cardiomyopathy.

## References

[1] Civelek T., Baser D.F., Saritas Z.K., Varol K. (2012). Case report: Exercise induced cardiac hypertrophy in a dog. Rev. Méd. Vét..

[2] Santos P.O.P.R., Santos E.A., Reis A.C., Santos A.M.M.R., Kuster M.C.C., Trivilin L.O., Aptekmann K.P. (2018). Effect of exercise on cardiovascular parameters in search and rescue-trained dogs. Arq. Bras. Med. Veterinária Zootec..

[3] Stepien R.L., Hinchcliff K.W., Constable P., Olson J. (1998). Effect of endurance training on cardiac morphology in Alaskan sled dogs. J. Appl. Physiol..

[4] Shave R., Howatson G., Dickson D., Young L. (2017). Exercise-Induced Cardiac Remodeling: Lessons from Humans, Horses, and Dogs. Vet. Sci..

[5] Kim J., Williams F.J., Dreger D.L., Plassais J., Davis B.W., Parker H.G., Ostrander E.A. (2018). Genetic selection of athletic success in sport-hunting dogs. Proc. Natl. Acad. Sci. USA.

[6] Chiacchio M., Omar A., Suau R. (2010). Respuesta de la tensión arterial a la prueba de esfuerzo. Apunt. Med. Esport..

[7] Espinosa J.S., Sánchez-Lafuente C. (2002). Prueba de Esfuerzo Cardíaca, Respiratoria y Deportiva.

[8] Rovira S., Muñoz A., Riber C., Benito M. (2010). Heart rate, electrocardiographic parameters and arrhythmias during agility exercises in trained dogs. Rev. Méd. Vét..

[9] Hamlin R.L., Smith C.R. (2006). Categorization of common domestic mammals based upon their ventricular activation process. Ann. N. Y. Acad. Sci..

[10] Constable P., Hinchcliff K., Olson J.L., Stepien R.L. (2000). Effects of endurance training on standard and signal-averaged electrocardiograms of sled dogs. Am. J. Vet. Res..

[11] Schneider H.P., Truex R.C., Knowles J.O. (1964). Comparative observations of the hearts of mongrel and Greyhound dogs. Anat. Rec. Adv. Integr. Anat. Evol. Boil..

[12] Strohm L., Visser L.C., Chapel E., Drost W., Bonagura J. (2018). Two-dimensional, long-axis echocardiographic ratios for assessment of left atrial and ventricular size in dogs. J. Vet. Cardiol..

[13] Boon J., Wingfield W.E., Miller C.W. (1983). Echocardiographic indices in the normal dog. Vet. Radiol..

[14] Bavegems V., Duchateau L., Sys S.U., De Rick A. (2007). Echocardigraphic reference values in whippets. Vet. Radiol. Ultrasound.

[15] Gugjoo M.B., Hoque M., Saxena A.C., Zama M.M.S., Dey S. (2014). Reference values of M-mode echocardiographic parameters and indices in conscious Labrador Retriever dogs. Iran. J. Vet. Res..

[16] Giraut S., Häggström J., Koskinen L.L.E., Lohi H., Wiberg M. (2019). Breed-specific reference ranges for standard echocardiographic measurements in salukis. J. Small Anim. Pr..

[17] Bodh D., Hoque M., Saxena A.C. (2019). Echocardiographic study of healthy Indian Spitz dogs with normal reference ranges for the breed. Vet. World.

[18] Kosić L.S., Trailović D.R., Krstić N. (2017). Age-dependent electrocardiographic and echocardiographic changes in German Shepherd dogs. Iran. J. Vet. Res..

[19] Visser L.C., Scansen B.A., Schober K.E., Bonagura J.D. (2015). Echocardiographic assessment of right ventricular systolic function in conscious healthy dogs: Repeatability and reference intervals. J. Vet. Cardiol..

[20] Jacobson J.H., Boon J.A., Bright J. (2013). An echocardiographic study of healthy Border Collies with normal reference ranges for the breed. J. Vet. Cardiol..

[21] Kurosawa T., Sist M., Sanders R.A. (2018). Echocardiographic variables in healthy North American Salukis. J. Vet. Cardiol..

[22] Vörös K., Hetyey C., Reiczigel J., Czirok G.N., Vȍrȍs K. (2009). M-mode and two-dimensional echocardiographic reference values for three Hungarian dog breeds: Hungarian Vizsla, Mudi and Hungarian Greyhound. Acta Vet. Hung..

[23] Page A., Edmunds G., Atwell R. (1993). Echocardiographic values in the Greyhound. Aust. Vet. J..

[24] Della Torre P.K., Kirby A.C., Church D.B., Malik R. (2000). Echocardiographic measurements in greyhounds, whippets and Italian greyhounds--dogs with a similar conformation but different size. Aust. Vet. J..

[25] Martin B.B., Reef V.B., Parente E.J., Sage A.D. (2000). Causes of poor performance of horses during training, racing, or showing: 348 cases (1992–1996). J. Am. Vet. Med. Assoc..

[26] Holler P., Wess G. (2013). Sphericity Index and E-Point-to-Septal-Separation (EPSS) to Diagnose Dilated Cardiomyopathy in Doberman Pinschers. J. Vet. Intern. Med..

[27] Calvert C., Meurs K.M., Bonagura J. (2000). Doberman Pinschers occult cardiomyopathy. Kirk’s Current Veterinary Therapy.

[28] Boraita A. (2019). Fatiga cardíaca en los deportistas: Una realidad en la que hay que pensar. Rev. Argent. Cardiol..

[29] Boraita A., Baño A., Berrazueta J.R., Lamiel R., Luengo E., Manonelles P., Pons I.C. (2001). Guidelines of the Spanish Society of cardiology for physical activity in patients with cardiac disease. Arch. Med. Deporte.

[30] Boraita A., Heras M.-E., Morales F., Marina-Breysse M., Canda A., Rabadan M., Barriopedro M.I., Varela A., De la Rosa A., Tuñón J. (2016). Reference values of the Aorta in elite athletes. Circ. Cardiovasc. Imaging.

[31] Rishniw M., Caivano D., Dickson D., Vatne L., Harris J., Matos J.N. (2019). Two-dimensional echocardiographic left-atrial-to-aortic ratio in healthy adult dogs: A re-examination of reference intervals. J. Vet. Cardiol..

